# Supplementary material for: Redox cycling of sulfur via microbes in soil boosts the bioavailability of nutrients to Brassica napus
Source: PLoS One. 2025 Feb 24;20(2):e0318936. doi: 10.1371/journal.pone.0318936 (PMC11849842; doi:10.1371/journal.pone.0318936)
Supplement: Attachment — Submitted filename: PONE-D-24-23262 (1).pdf [file pone.0318936.s001.pdf]

PLOS ONE

Redox cycling of sulfur via microbes in soil boosts the bioavailability of nutrients to Brassica napus

--Manuscript Draft--

Manuscript Number:	PONE-D-24-23262
Article Type:	Research Article
Full Title:	Redox cycling of sulfur via microbes in soil boosts the bioavailability of nutrients to Brassica napus
Short Title:	Microbial Sulfur Cycling Enhances Nutrient Uptake in Brassica napus
Corresponding Author:	Tajwar Alam, Ph.D<br>Pir Mehr Ali Shah Arid Agriculture University: PMAS-Arid Agriculture University Rawalpindi<br>Rawalpindi, Pakistan PAKISTAN
Keywords:	Sulfur oxidizing bacteria; hydrogen sulfide; sulfur reducing bacteria; bacterial consortium; canola
Abstract:	Deficiency of sulfur (S) has been recognized for both dry and wetland plants as a significant growth limiting factor. S-oxidizers enhance the oxidation rate of S and increase sulfate (SO_4^{-2}) production by making them available in critical growth stages, resulting in higher plant growth and yield. A two-phase study was undertaken to assess the effectiveness of microbial redox reactions on soil nutrient bioavailability and crop growth. In the first phase isolation of S bacteria was carried out while in the second phase, a pot experiment was conducted and these bacteria were evaluated as a nutrient source along with different ratios of NPK fertilizer by growing canola (Brassica napus) as a test crop. Treatment combinations were, viz., Control (no inoculum), ½NPK fertilizer (50, 30, and 25 kg ha^{-1}), Full NPK fertilizer (100, 60, and 50 kg ha^{-1}), ½NPK+SOB, ½ NPK+SRB and ½NPK+SOB+SRB. Sulfur oxidizing bacteria (SOB) strains were isolated from samples and then screened based on pH reduction (in thiosulphate broth). Sulfur-reducing bacteria (SRB) are characterized by their anaerobic energy metabolism based on the dissimilatory reduction of SO_4^{-2} to hydrogen sulfide (H_2S) or S° to be useful for SOB. Among different bacterial consortiums, the best results for nutrient bioavailability (macro- and micronutrient) in soil and plant was shown by (½NPK+SOB+SRB) followed by (½NPK+SOB). The highest values of chemical parameters were observed in (½NPK+SOB+SRB) followed by (½NPK+SOB). The highest pH reduction was shown by (½NPK+SOB) followed by (½NPK+SOB+SRB). This study demonstrates that microbial inoculation along with ½NPK increases the bioavailability of macro- and micronutrients for crops.
Order of Authors:	Rabia Aslam
	Ghulam Jilani
	Tajwar Alam, Ph.D
	Zia Ul Haq
	Muhammad Ikram
	Nida Fatima
Additional Information:	
**Question**	**Response**
**Financial Disclosure**<br><br>Enter a financial disclosure statement that describes the sources of funding for the work included in this submission. Review the submission guidelines for detailed requirements. View published research	The author(s) received no specific funding for this work.

articles from *PLOS ONE* for specific examples.<br><br>This statement is required for submission and **will appear in the published article** if the submission is accepted. Please make sure it is accurate.<br><br>**Funded studies**<br>Enter a statement with the following details:<br>• Initials of the authors who received each award<br>• Grant numbers awarded to each author<br>• The full name of each funder<br>• URL of each funder website<br>• Did the sponsors or funders play any role in the study design, data collection and analysis, decision to publish, or preparation of the manuscript?<br><br>Did you receive funding for this work?	
**Competing Interests**<br><br>Use the instructions below to enter a competing interest statement for this submission. On behalf of all authors, disclose any competing interests that could be perceived to bias this work—acknowledging all financial support and any other relevant financial or non-financial competing interests.<br><br>This statement is required for submission and **will appear in the published article** if the submission is accepted. Please make sure it is accurate and that any funding sources listed in your Funding Information later in the submission form are also declared in your Financial Disclosure statement.<br><br>View published research articles from *PLOS ONE* for specific examples.	The authors declare that they have no known competing financial interests or personal relationships that could have appeared to influence the work reported in this paper

Powered by Editorial Manager® and ProduXion Manager® from Aries Systems Corporation

**NO authors have competing interests**<br><br>Enter: *The authors have declared that no competing interests exist.*<br><br>**Authors with competing interests**<br><br>Enter competing interest details beginning with this statement:<br><br>*I have read the journal's policy and the authors of this manuscript have the following competing interests: [insert competing interests here]*<br><br>* typeset	
**Ethics Statement**<br><br>Enter an ethics statement for this submission. This statement is required if the study involved:<br><br>• Human participants<br>• Human specimens or tissue<br>• Vertebrate animals or cephalopods<br>• Vertebrate embryos or tissues<br>• Field research<br><br>Write "N/A" if the submission does not require an ethics statement.<br><br>General guidance is provided below. Consult the submission guidelines for detailed instructions. **Make sure that all information entered here is included in the Methods section of the manuscript.**	N/A

Powered by Editorial Manager® and ProduXion Manager® from Aries Systems Corporation

**Format for specific study types**<br><br>**Human Subject Research (involving human participants and/or tissue)**<br>• Give the name of the institutional review board or ethics committee that approved the study<br>• Include the approval number and/or a statement indicating approval of this research<br>• Indicate the form of consent obtained (written/oral) or the reason that consent was not obtained (e.g. the data were analyzed anonymously)<br><br>**Animal Research (involving vertebrate animals, embryos or tissues)**<br>• Provide the name of the Institutional Animal Care and Use Committee (IACUC) or other relevant ethics board that reviewed the study protocol, and indicate whether they approved this research or granted a formal waiver of ethical approval<br>• Include an approval number if one was obtained<br>• If the study involved *non-human primates*, add *additional details* about animal welfare and steps taken to ameliorate suffering<br>• If anesthesia, euthanasia, or any kind of animal sacrifice is part of the study, include briefly which substances and/or methods were applied<br><br>**Field Research**<br><br>Include the following details if this study involves the collection of plant, animal, or other materials from a natural setting:<br>• Field permit number<br>• Name of the institution or relevant body that granted permission	
**Data Availability**<br><br>Authors are required to make all data underlying the findings described fully available, without restriction, and from the time of publication. PLOS allows rare exceptions to address legal and ethical concerns. See the PLOS Data Policy and FAQ for detailed information.	Yes - all data are fully available without restriction

Powered by Editorial Manager® and ProduXion Manager® from Aries Systems Corporation

A Data Availability Statement describing where the data can be found is required at submission. Your answers to this question constitute the Data Availability Statement and **will be published in the article**, if accepted.<br><br>**Important:** Stating 'data available on request from the author' is not sufficient. If your data are only available upon request, select 'No' for the first question and explain your exceptional situation in the text box.<br><br>Do the authors confirm that all data underlying the findings described in their manuscript are fully available without restriction?	
**Describe where the data may be found in full sentences. If you are copying our sample text, replace any instances of XXX with the appropriate details.**<br><br>• If the data are **held or will be held in a public repository**, include URLs, accession numbers or DOIs. If this information will only be available after acceptance, indicate this by ticking the box below. For example: *All XXX files are available from the XXX database (accession number(s) XXX, XXX.).*<br>• If the data are all contained **within the manuscript and/or Supporting Information files**, enter the following: *All relevant data are within the manuscript and its Supporting Information files.*<br>• If neither of these applies but you are able to provide **details of access elsewhere**, with or without limitations, please do so. For example:<br><br>*Data cannot be shared publicly because of [XXX]. Data are available from the XXX Institutional Data Access / Ethics Committee (contact via XXX) for researchers who meet the criteria for access to confidential data.*<br><br>*The data underlying the results presented in the study are available from (include the name of the third party*	All relevant data are within the manuscript and its Supporting Information files.

and contact information or URL).<br>• This text is appropriate if the data are owned by a third party and authors do not have permission to share the data.<br><br>* typeset	
Additional data availability information:	

Powered by Editorial Manager® and ProduXion Manager® from Aries Systems Corporation

Manuscript

Click here to access/download;Manuscript;Rabia MS for PLOS ONE.docx

Redox cycling of sulfur via microbes in soil boosts the bioavailability of nutrients to *Brassica napus*

Rabia Aslam[1], Ghulam Jilani[1*], Tajwar Alam[1,2*], Zia-Ul-Haq[3], Muhammad Ikram[1], Nida Fatima[1],

[1]Institute of Soil and Environmental Sciences, PMAS Arid Agriculture University, Rawalpindi 46300, Pakistan

[1,2]Institute of Hydroponic Agriculture, PMAS Arid Agriculture University, Rawalpindi 46300, Pakistan

[3]Faculty of Agriculture Engineering, PMAS-Arid Agriculture University, Rawalpindi 46300, Pakistan

Corresponding Authors

tajwaralam@uaar.edu.pk

jilani@uaar.edu.pk

Abstract

Deficiency of sulfur (S) has been recognized for both dry and wetland plants as a significant growth limiting factor. S-oxidizers enhance the oxidation rate of S and increase sulfate (SO_4^{-2}) production by making them available in critical growth stages, resulting in higher plant growth and yield. A two-phase study was undertaken to assess the effectiveness of microbial redox reactions on soil nutrient bioavailability and crop growth. In the first phase isolation of S bacteria was carried out while in the second phase, a pot experiment was conducted and these bacteria were evaluated as a nutrient source along with different ratios of NPK fertilizer by growing canola (*Brassica napus*) as a test crop. Treatment combinations were, viz., Control (no inoculum), ½NPK fertilizer (50, 30, and 25 kg ha^{-1}), Full NPK fertilizer (100, 60, and 50 kg ha^{-1}), ½NPK+SOB, ½ NPK+SRB and ½NPK+SOB+SRB. Sulfur oxidizing bacteria (SOB) strains were isolated from samples and then screened based on pH reduction (in thiosulphate broth). Sulfur-reducing bacteria (SRB) are characterized by their anaerobic energy metabolism based on the dissimilatory reduction of SO_4^{-2} to hydrogen sulfide (H_2S) or S° to be useful for SOB. Among different bacterial consortiums, the best results for nutrient bioavailability (macro- and micronutrient) in soil and plant was shown by (½NPK+SOB+SRB) followed by (½NPK+SOB). The highest values of chemical parameters were observed in (½NPK+SOB+SRB) followed by (½NPK+SOB). The highest pH reduction was shown by (½NPK+SOB) followed by (½NPK+SOB+SRB). This study demonstrates that microbial inoculation along with ½NPK increases the bioavailability of macro- and micronutrients for crops.

KEYWORDS: Sulfur oxidizing bacteria; hydrogen sulfide; sulfur reducing bacteria; bacterial consortium; canola

Introduction

Sulfur is among the essential nutrients required for the proper growth of plants, animals, humans, and microorganisms [1]. Being a key component of amino acids (methionine, cysteine, and cystine) S facilitates the formation of additional S-reducing complexes having dynamic functions in the development of enzymes and proteins [2]. Sulfur plays a key role in the formation of vitamins, proteins, and oils improving compounds in plants. Deficiency of S results in lower photosynthetic activity, reduced metabolism of nitrogen (N), lower oil content, and growth impedance in plants with significant effects on shoots and root growth [3]. Its deficiency causes a decrease in S-containing amino acids and protein synthesis [4]. Additionally, its deficiency causes the yellowing of younger leaves known as chlorosis followed by necrosis in later developmental stages. Lower S availability also affects N fixation because both N and S are central parts of the protein [5]. A higher quantity of S is required by oilseed crops, viz., soybean, groundnut, rapeseed, and sunflower followed by cereals and pulses [6]. Therefore, to increase S availability, S-oxidizers are used to improve the natural oxidation rate and enhance the production of SO_4^{-2}, making it available to crop plants at critical stages [7]. The application of mineral fertilizer having S has also been reported to improve nutrient availability, by improving soil physicochemical properties [8].

Application of S increased the synthesis of amino acids and also enhanced amounts of N_2 fixed in leguminous plants and soil [9]. The availability of S in the soil is affected by physiochemical factors and pedogenic processes. Most soils across the globe are S-deficient [10]. Sulfur deficiency is more common compared to other nutrients in soils of Northern Europe with oilseed cropping [11]. Additionally, S deficiency could also be attributed to decreased S storage via deposition from the atmosphere in the last two to three decades. Furthermore, S-containing fertilizers like single superphosphate, farmyard manure, and compost have been substituted by chemical fertilizers having no or little S [12].

Beneficial microorganisms from the rhizosphere and phyllo-sphere were isolated and evaluated as plant growth promotors to reduce agrochemicals application in soil [2]. Mechanisms directly involved in plant growth promotion relate to higher nutrient acquisition through fixation, solubilization in soil (N fixation, P, K, and S solubilization), hormone production (auxins, cytokinin, aminolevulinic acid, gibberellins, and abscisic acid) and iron sequestration through

bacterial siderophores, and ACC deaminase synthesis to reduce formation of ethylene [13]. Whereas, indirect growth stimulation mechanisms include, the reduction of stresses viz., salinity, drought, heavy metals, and temperature [14]. Microbes possessing plant growth-promoting potential have been commercialized as biofertilizers, viz., N-fixing, P-solubilizing, K-mobilizers, PGPR, and mycorrhizal fungi [15], and inoculation of such microbes alter microbial diversity and change rooting patterns that help in nutrients management [16]. Microbes involved in S cycling could be used as bio-fertilizers, having low-input and environment-friendly technology for sustainable agriculture ecosystems [17].

Plants absorb S in the form of inorganic SO_4^{2-} and some microbes can oxidize S into SO_4^{-2} form known as sulfur-oxidizing bacteria (SOB) [18]. Bacteria belonging to genus Thiobacillus and Acidithiobacillus, are involved in S-oxidation (Shinde et al., 2022). Several studies have been undertaken by using SOB as a microbial inoculant and results showed around 47–69% of onion yield increase as compared to control [1]. Moreover, SOB inoculation and N-fixing bacteria combinedly improved plant yield and N uptake (220%, and 630%, respectively) compared to non-inoculated plants. Similarly, bio-fertilization has N-fixing strains (*Azotobacter* and *Azospirillum*), in addition to the inorganic N-enhanced oil content and grain yield of canola [19]. SOB enhanced S-oxidation resulting in higher availability of SO_4^{-2} to mustard crop [20].

Sulfur bacteria integrate a diverse group of organisms having the capability to share oxidized, reduced, or partially oxidized inorganic S compounds. Genus *Thiobacillus* is the most important organism in different groups of bacteria (SOB) which are responsible for S oxidization. Application of *Thiobacillus* bacteria enhances S and P availability in soil. Canola is the most essential oil seed crop across the globe [21]. P-solubilizing and SOB enhance canola efficiency in calcareous soils by improving the absorption of plant nutrients. Bacterial inoculant operations can be improved by the addition of organic matter (OM). Therefore, the correct combination of chemical and biological sources can boost canola production and development considerably by improving nutrient absorption [22]. Keeping in view the S importance as a key macronutrient, further studies are needed on soil microorganisms involved in their biogeocycle.

Current study objective was to evaluate the impact of the recommended dose of NPK with or without SOB and the combined effects of SOB and SRB along with the recommended

dose of NPK. It was inferred that their integrated application synergistically affects macro- and micronutrient bioavailability and uptake by canola in soil.

2 Materials and Methods

2.1 Soil characterization

Ten composite samples of soil (0–30 cm) were collected at the start of the experiment to analyze soil pH, EC [23], OM [24], and texture [25]. The texture of experimental soil was recorded as silty clay loam, having pH = 7.53, EC = 0.252 dSm^{-1}, N (0.54 g kg^{-1}), P (6.91 mg kg^{-1}), K (131 mg kg^{-1}), S (6.94 mg kg^{-1}) zinc (0.31 mg kg^{-1}), manganese (4.01 mg kg^{-1}), and iron (4.2 mg kg^{-1}).

2.2 Isolation of SOB and SRB

SOB isolation was carried out with a thiosulphate broth medium. Its composition was, viz., $Na_2S_2O_3$, $NaHCO_3$, 0.2 g; 5.0 g; 0.1 g; NH_4Cl, K_2HPO_4, 0.1 g dissolved in distilled (DI) water (DI) (1.0 L). Medium pH was adjusted to 8.0 and Bromocresol purple was used as indicator. The medium was autoclaved for sterilization and subsequently poured into pre-sterilized tubes and upon condensation, the streaking was done to purify the isolated strains. The tubes were incubated for 4-5 days at 30°C [26].

Enrichment and isolation of SRB were done by using a medium containing DI water per liter: ammonium sulfate 5.3 g, sodium-acetate 2.0 g, KH_2PO_4 0.5 g, magnesium sulfate.$7H_2O$ 0.2 g, sodium chloride 1.0 g, calcium chloride. $2H_2O$ 0.1 g. Solution: 1 10.0 mL and Solution: 2 1.0mL. Solution one having per liter of DI water: Nitrilotriacetic acid 12.8 g, $FeCI_2.4H_2O$ 300.0 mg, copper chloride 20.0 mg, $MnCI_2.4H_2O$ 100.0 mg, $COCI_2.6H_2O$ 170 mg, zinc chloride 100 mg, $H_3BO_3$10.0 mg, $Na_2MoO_4.2H_2O$ 10 mg. Solution two has DI water 100 mL Resazurin 0.2 g. Followed by autoclave and cooling, the medium was provided with anaerobic sterile stock solutions from components: 50 mL of 8% Na_2CO_3 water. 5.5 mL of 25% hydrochloric acid. About 1.0 mL of 8.7% $Na_2S_2O_4$ in water and, pH was adjusted to 7.2 by adding HCI [27].

2.3 Purification of SOB and SRB

Isolate purification was carried out by moving the isolates to the medium of the new

broth. The streaking of isolates was carried out on thiosulfate ($S_2O_3^{2-}$) agar plates to obtain individual colonies. For characterization and further testing, these pure isolates have been maintained [28].

2.4 Characterization of SOB and SRB

Isolated strain characterization was done through colony morphology, elevation pattern, colony margins, colony colour, colony form, and opacity. Additionally, biochemical and morphological characteristics were studied to characterize isolated bacterial strains by following [29].

2.5 Gram Staining

Bacterial strains were further subjected to gram staining as explained by [30]. Wire loop was first heated on a spirit lamp then it was full of individual isolated bacterial strains that were spread on a glass slide followed by air-drying and stained by using crystal violet for two minutes followed by slight washing with deionized water. Later on, the smear was flooded with iodine solution and de-colorized by using 75% alcohol. After de-colorization, the smear was stained with safranin. The smear was dried by passing a glass slide 2 to 3 times from spirit lamb and the slide was placed under a light microscope for observation of the staining reaction of individual isolates.

2.6 Treatment plan and experimental design

A greenhouse experiment was undertaken at PMAS-Arid Agriculture University Rawalpindi, to assess the potential impact of inoculation of SOB and SRB, and synthetic fertilizer on canola production, soil nutrients (macro- and micronutrient) bioavailability, and plant uptake. Soil was collected from the university research area and pots were filled (8 kg each) with air-dried and sieved soil (2 mm). The treatment combination was control, NPK half dose (½NPK) (50–30-25 mg kg^{-1}), full dose of NPK (100–60-50 mg kg^{-1}), ½NPK + SOB, ½ NPK + SRB, and ½ NPK + SOB+ SRB. A completely randomized design (CRD) was implemented with three replications. Treatments encompassing bacteria and synthetic fertilizer were added to the soil before the sowing of the canola crop. Synthetic fertilizer (NPK) was applied as a basic dose before sowing as DAP, urea, and KSO_4^{-2}, in all pots. Sterilized DI water was used to dilute bacterial inoculums

at the rate of 1% v/v. Ten seeds of canola (*Brassica napus*) per pot were sown in November 2021. After seedlings establishment, thinning of plants was done (five plants^{-1}). Soil moisture (70–80%) was maintained and weeds were manually removed wherever required.

2.7 Crop harvesting

Harvesting of canola was done after 145 days of sowing. Different attributes, viz., shoot length, fresh shoot - and dry weight, and root fresh- and dry weight were recorded. The shoot and root dry weight of each plant was determined by separating roots from shoots with DI water and stored in an oven at 65±3 °C for 3 days.

2.8 Analysis of soil

To evaluate the impact of bacterial inoculates and synthetic fertilizer on S and other elements, post-harvest soil was collected and analyzed for total-N [31], AB-DTPA available-P, and extractable-K [32]. AB-DTPA extractable-Mn, Zn, and Fe were recorded by following [33]. Soil texture was measured through the hydrometer method [25]. Soil pH and EC were measured through soil pH and EC meter [23]. Soil organic matter was analyzed by following [34].

2.9 Plant analysis

Canola plant leaves were harvested at the maturity stage, and dried in the oven at 65±3 °C for 3 days, and dry weight was noted. Dried shoots/leaves were ground to powder and dry ashing was done up to 4 h at 550 °C through a muffle furnace. Digestate was used to analyze Mn, Fe, and Zn at different wavelengths, 279.5 nm, 248.7 nm, and 213.7 nm respectively, and analyzed by using an atomic absorption spectrophotometer. Potassium contents were analyzed through a flame photometer. Phosphorous contents were measured by following [35]. Kjeldahl method was used to measure total-N [31].

2.10 Statistical analysis

All data was analyzed by using Statistix 8.1. ANOVA and multiple comparison analyses were performed using the Tukeys test at $P<0.05$. Means were compared by using the least significant difference test ($LSD_{0.05}$) for treatments' statistical significance.

3 Results

3.1 SOB and SRB isolate attributes

Isolated S-oxidizing bacteria was *Thiobacillus thiooxidans*, a gram-negative chemo-lithotroph bacteria. They utilize $S_2O_3^{2-}$ and sulfide as energy sources to produce sulphuric acid. These are aerobic sulfur bacteria and they derive energy from the oxidation of sulfide or elemental sulfur (S^0) to SO_4^{-2}. The isolated S-reducing bacteria was Desulfvibrio vulgaris, Gram-negative, non-spore-forming, anaerobic, curved rod-shaped PGPR.

3.2 Growth attributes of canola

Plant attributes like root, shoot, and plant biomass of canola differed significantly with bacterial inoculation in soil. Results indicated that inoculation of SOB and SRB enhanced canola biomass compared with sole NPK application. The highest shoot length of 100 cm and the highest root length of 26.8 cm were noted in T6 (½NPK+SOB+SRB) (Table 1). The highest fresh root weight (g plant^{-1}) of 2.58 was recorded in T5 (½NPK+SRB), while the highest fresh shoot weight (g plant^{-1}) of 28.6 was noted in T6 (½NPK+SOB+SRB). The highest dry root weight (g plant^{-1}) of 2.03 was noted in T6 (½NPK+SOB+SRB), while the highest dry shoot weight (g plant^{-1}) of 15.9 was obtained in T6 (½NPK+SOB+SRB) (Table 1).

3.3 Nutrient contents in canola

The concentration of nutrients in plant tissue at maturity depicted a significant response to applied treatments. The highest total-N (1.53%), P (2.80%) concentration was recorded in T6 (½NPK+SOB+SRB) while the highest K (1.49%) concentration was recorded in T2 (full NPK) as reported in Table 2. The highest total-S (0.21%) was recorded in T6 (½NPK+SOB+SRB) followed by treatment 1/2 NPK+SOB (0.18%) while the lowest total-S (0.043%) was recorded for the control treatment (Table 2). A blend of ½NPK synthetic fertilizer with SOB and SRB (T6) improved Mn significantly (0.06 g kg^{-1}), Fe (0.023 g kg^{-1}), Zn (0.045 g kg^{-1}), and Cu (0.092 g kg^{-1}) contents in plants tissue as depicted in Table 3. Inoculation of SOB and SRB with half NPK, improved nutrient contents in canola crops significantly, which suggests these microorganisms' role in nutrient mobilization.

3.4 Post-harvest soil pH, EC, and OM contents

Post-harvested soil amended through bacteria significantly affected pH and OM. The decreasing pattern was shown in pH with various applied treatments, ranging from 7.5 in the control treatment to 7.1 in T4 (1/2NPK+SOB) closely followed by T6 (7.2) 1/2NPK+SOB+SRB (Table 4). However, OM and EC were increased slightly by different treatment applications. Soil OM content was recorded at 0.50% in control and 0.61% in treatment T6 (1/2 NPK+SOB+SRB). Soil EC was increased from 0.251 dS m^{-1} in T1 (control) treatment to 0.492 dS m^{-1} in T6 (1/2NPK+SOB+SRB) (Table 4).

3.5 Nutrient contents in post-harvest soil

Extractable-P concentration significantly improved over control (0.057 g kg^{-1}) with treatments applied up to the highest rate of 0.025 g kg^{-1} at T6 (½NPK+SOB+SRB). Soil extractable-K and total-N also improved. Total-N varied between 0.56% to 1.53% showing that total-N was slightly short in post-harvest soil. Soil extractable-K varied from 1.31 % to 1.56 %. Soil S ranged from 6.73% to 13.3% (Table 5). Compared to control significant improvement in soil Cu, Mn, Fe, and Zn was recorded in treatment T6 (½NPK+SOB+SRB) with highest values of 0.32, 1.49, 2.54, and 1.33 mg kg^{-1}, respectively (Table 6).

3.6 Discussion

Present study reinforced the above-stated hypothesis and revealed that the application of SOB and SRB along with chemical fertilizer highly influenced physicochemical attributes of soil, enhanced canola growth, and increased bioavailability of nutrient contents in soil. Findings of the current study are in accordance with literature published previously [36], which reported that improvement in crop parameters is because of the release of bacterial metabolite and nutrient mineralization. The increase in crop shoot-root length and plant biomass was owing to the production of exopolysaccharides, siderophores, and phytohormones, and enzyme activation by *Leptothrix discophora* and *Bacillus polymyxa* [37]. Biofertilizer application enhances the growth of plants by improving the availability of nutrients in the rhizosphere through the production of antibiotics and by hindering pathogenic bacteria growth [38].

Inoculation of SOB and SRB with synthetic fertilizer significantly improved plant growth and yield attributes of canola compared to control [39]. Our findings also revealed that nutrient

augmentation was much higher when the SOB and SRB inoculants were applied with half NPK recommended dose. Application of Bacillus spp. improved micronutrients, viz., Mn, Zn, and Fe in plants [40]. Soil microbes contribute to solubility and henceforth improve soil micronutrients [41]. The impact of PGPR on plant production is well documented and has been attributed to the synthesis of phytohormone and a greater supply of nutrients [42]. Microbes use several methods to enhance the solubility of nutrients in soil like as altering plant metabolism and changing root exudates [43-44]. Microbial inoculation of *Bacillus mucilaginous* and *Bacillus megaterium* improved plant growth [45].

Oxidation and reduction of sulfur by microbes are the most active processes in the S-cycle carried out by SOB and SRB and are considered vital phenomena in S biogeochemical cycling. Generally, on a nutritional basis, SRB and SOB are characterized as litho-autotrophs. Reduced compounds of S are oxidized by SOB like H_2S, S^0, sulfite (SO_3^{-2}), $S_2O_3^{2-}$, and SnO_6^{2-} or $-SnO_6^{-}$ into SO_4^{-2}. However, SO_4^{-2} serves as an SRB electron acceptor in anaerobic conditions and reduces SO_4^{-2} and other S compounds ($S_2O_3^{2-}$, SO_3^{-2}, S^0) into H_2S. Moreover, in a natural ecosystem, SRB reduces SO_4^{-2} through assimilatory and dissimilatory reactions. SRB utilizes various types of enzymes in dissimilatory reactions to reduce S substrate, while SO_4^{-2} is assimilated in organic compounds via an assimilatory process through S substrate reduction. Soil used in the present study was slightly calcareous; both SOB (*Thiobacillus thiooxidans*) and SRB (*Desulfvibrio vulgaris*) inoculation significantly reduced soil pH. This might be because of organic acid production in the rhizosphere by soil microorganisms. Bacteria produce the few organic acids in soil called carboxylic acids [46], which lower soil pH in the rhizosphere and dissociate calcium phosphate bounds in calcareous soils. Furthermore, microbes modify redox potential and surrounding medium pH [47]. Our results are also in line with [48], who stated that a living organism’s presence in soil produces pH variation and momentous redox potential within the soil. SOB or SRB has an important role as they significantly affect pH and redox potential [47]. Plant root's exudation of protons (H+), carboxylates, and enzymes also affects soil pH [49]. SOB and SRB improve the fertility of the soil by regulating pH and EC. During this study, microbial inoculate application slightly increased OM content of the soil. *Bacillus polymyxa* and *Thiobacillus thiooxidans* increase OM in soil via the release of numerous exopolysaccharides that break down large polymeric substances which in turn offer nutrients to plants [50].

Nutrient availability is controlled by pH and redox conditions [51]. Additionally, soil microfauna is also a key factor for nutrient dynamics in the soil [47]. We noted that soil post-harvest nutrients (macro- and micro) responded greatly to SOB and SRB inoculation in combination with 1/2NPK fertilizer. The highest uptake of nutrients by plants was recorded at pH 7.0, even with the lowest chemical fertilizer dose. Similar results have been reported by other researchers. According to [52], rhizosphere acidification and salinization are the most significant factors affecting the availability of nutrients in soil. Inoculation of seeds with *Leptothrix discophora* and *Bacillus polymyxa* bacteria enhanced solubilization as well as mineralization of macronutrients [53]. Several bacterial species play a vital role in increasing soil fertility through increasing OM, which enhances the availability of macronutrients in soil [1]. Moreover, by producing organic acids they contribute to nutrient mobilization and uptake by plants. The population of SOB and SRB is a key factor in the availability of soil S for plants [54]. Microorganisms mobilize S in aerated soils via SOx reduction that promotes H+ root excretion, decreases the pH of saline soils, and improves the availability of micronutrients [1]. Availability of soil S is affected by the pH of the soil and improves greatly owing to decreases in pH. It could be due to S oxides becoming stronger oxidants when the pH of soil decreases, resulting in more easily reduced S ions [55].

Conclusion

Findings of the current study revealed that the application of bacteria (S-oxidizing and S-reducing) with 1/2NPK fertilizer rendered higher S availability as well as nutrients to canola by decreasing S immobilization in soil. Furthermore, significant improvement in the fertility status of soil was noted. In conclusion, these results suggest that combined application of synthetic fertilizers along with SOB and SRB inoculation as a soil amendment improves plant growth attributes, and nutrients in plants and soil. It is easily adaptable by farmers and eco-friendly methods to reduce crop nutrient rations, high-yield production, and sustain satisfactory profit.

Additional information

No additional information is available for this paper.

Acknowledgment

This research article is based on the MS thesis research of Rabia Aslam. We are thankful to the Institute of Soil & Environmental Sciences, PMAS Arid Agriculture University, Rawalpindi, Pakistan, for providing greenhouse and laboratory facilities for experimentation and analysis.

References

1. Chaudhary, S., Sindhu, S. S., Dhanker, R., & Kumari, A. (2023). Microbes-mediated sulphur cycling in soil: Impact on soil fertility, crop production and environmental sustainability. Microbiological Research, 127340.
2. Khuong, N. Q., Kantachote, D., Dung, N. T. T., Huu, T. N., Thuc, L. V., Thu, L. T. M., ... & Xuan, L. N. T. (2023). Potential of potent purple nonsulfur bacteria isolated from rice-shrimp systems to ameliorate rice (Oryza sativa L.) growth and yield in saline acid sulfate soil. Journal of Plant Nutrition, 46(3), 473-494.
3. Divito, G. A., Echeverría, H. E., Andrade, F. H., & Sadras, V. O. (2015). Diagnosis of S deficiency in soybean crops: performance of S and N: S determinations in leaf, shoot and seed. Field Crops Research, 180, 167-175.
4. El-Lateef, A., Mohamed, E., El-Salam, A., El-Sayed, M., & Yousef, A. R. M. (2023). Impact of Legume Ameliorating with Sulphur and Phosphorus on seed Protein, S-Amino Acids and P Content. Egyptian Journal of Chemistry, 66(13), 1545-1553.
5. Almeida, L. F. A., Correndo, A., Ross, J., Licht, M., Casteel, S., Singh, M., & Ciampitti, I. A. (2023). Soybean yield response to nitrogen and sulfur fertilization in the United States: contribution of soil N and N fixation processes. European Journal of Agronomy, 145, 126791.
6. Borpatragohain, P., Rose, T. J., Liu, L., Barkla, B. J., Raymond, C. A., & King, G. J. (2019). Remobilization and fate of sulphur in mustard. Annals of Botany, 124(3), 471-480.
7. Stamford, N. P., Santos, P. R. D., Moura, A. M. M. F. D., & Freitas, A. D. S. D. (2003). Biofertilizers with natural phosphate, sulphur and Acidithiobacillus in a siol with low available-P. Scientia Agricola, 60(4), 767-773.
8. Malik, M. A., Khan, K. S., Marschner, P., & Ali, S. (2013). Organic amendments differ in their effect on microbial biomass and activity and on P pools in alkaline soils. Biology and Fertility of Soils, 49(4), 415-425.
9. Becana, M., Wienkoop, S., & Matamoros, M. A. (2018). Sulfur transport and metabolism in legume root nodules. Frontiers in plant science, 9, 1434.
10. Jadhav, S. C., Salvi, V. G., Kasture, M. C., More, S. S., & AA, W. (2022). Influence of different levels of Sulphur and biofertilizers on soil properties and yield of mulched

Groundnut (Arachis hypogaea L.) in lateritic soils of Konkan region. The Pharma Innovation Journal, 11(1), 1040-1045.

11. Naorem, A., Udayana, S. K., Kumar, G., & Mukherjee, A. K. (2017). Intervention of multitasking PGPR in pulse production under stress conditions. Innovative Farming, 2(1), 41-44.
12. Zhou, W., Wan, M., He, P., Li, S., & Lin, B. (2002). Oxidation of elemental sulfur in paddy soils as influenced by flooded condition and plant growth in pot experiment. Biology and fertility of soils, 36, 384-389.
13. Kumar, S., Sindhu, S. S., & Kumar, R. (2022). Biofertilizers: An ecofriendly technology for nutrient recycling and environmental sustainability. Current Research in Microbial Sciences, 3, 100094.
14. Du, M., Zhang, P., Wang, G., Zhang, X., Zhang, W., Yang, H., ... & Ma, F. (2022). H2S improves salt-stress recovery via organic acid turn-over in apple seedlings. Plant, Cell & Environment, 45(10), 2923-2942.
15. Basu, A., Prasad, P., Das, S. N., Kalam, S., Sayyed, R. Z., Reddy, M. S., & El Enshasy, H. (2021). Plant growth promoting rhizobacteria (PGPR) as green bioinoculants: recent developments, constraints, and prospects. Sustainability, 13(3), 1140.
16. Sindhu, S. S., Sehrawat, A., Phour, M., & Kumar, R. (2022). Nutrient acquisition and soil fertility: Contribution of rhizosphere microbiomes in sustainable agriculture. In Microbial BioTechnology for Sustainable Agriculture Volume 1 (pp. 1-41). Singapore: Springer Nature Singapore.
17. Gahan, J., O'Sullivan, O., Cotter, P. D., & Schmalenberger, A. (2022). Arbuscular Mycorrhiza Support Plant Sulfur Supply through Organosulfur Mobilizing Bacteria in the Hypho-and Rhizosphere. Plants, 11(22), 3050.
18. Shinde, A. H., Sharma, A., Doshi, S., Kumar, M. A., & Haldar, S. (2022). Isolation and screening of sulfur-oxidizing bacteria from coast of Bhavnagar, India, and formulation of consortium for bioremediation. Environmental Science and Pollution Research, 29(36), 54136-54149.
19. Namvar, A., & Khandan, T. (2015). Inoculation of rapeseed under different rates of inorganic nitrogen and sulfur fertilizer: impact on water relations, cell membrane

stability, chlorophyll content and yield. Archives of Agronomy and Soil Science, 61(8), 1137-1149.

20. Vinogradov, D. V., Naumtseva, K. V., & Lupova, E. I. (2019, October). Use of biological fertilizers in white mustard crops in the non-Chernozem zone of Russia. In IOP Conference Series: Earth and Environmental Science (Vol. 341, No. 1, p. 012204). IOP Publishing.
21. Stamford, N. P., Santos, P. R., Santos, C. E. S., Freitas, A. D. S., Dias, S. H. L., & Lira Jr, M. A. (2007). Agronomic effectiveness of biofertilizers with phosphate rock, sulphur and Acidithiobacillus for yam bean grown on a Brazilian tableland acidic soil. Bioresource Technology, 98(6), 1311-1318.
22. Zaeim, A. N., Torkaman, M., Ghasemeeyan, H., & Roohi, M. (2017). The Role of Bio Fertilizer in Canola Growth, Yield and Stress Condition.
23. Mclean, E. O. (1982). Soil pH and lime requirement. In: Page, A. L., Miller, R. H., &Keey, D. R. Eds. Methods of Soil Analysis Part 2. American Society of Agronomy, No.9. Madison, WI, USA., 199-209.
24. Olsen, S. R., & Sommers, L. E. (1982). Phosphorus. In: Page, A. L., Miller, R. H., &Keey, D. R. (Eds). Methods of Soil Analysis Part 2. Am. Soc. Agron. No. 9. Madison, Wisconsin, USA, 403-427.
25. Gee GW, Bauder JW (1986) Particle size analysis. In ‘Methods of soil analysis. Part1: Physical and mineralogical methods. 2nd edn (Ed. A Klute) pp. 383–411. Soil Science Society of America and American Society of Agronomy: Madison, WI, 435.
26. Beijerinck, M. W. (1904) Phenomenes de reduction proguits parles microbes Arch. Sci Exactes Nat Haarlem Ser 2:9131–9157. https://doi.org/10.12691/jaem-2-1-1
27. Badziong, W., & Thauer, R. K. (1978). Growth yields and growth rates of Desulfovibrio vulgaris (Marburg) growing on hydrogen plus sulfate and hydrogen plus thiosulfate as the sole energy sources. Archives of Microbiology., 117, 209-214.
28. Smibert RM, Kreig NR (1994) Phenotypic characterization In: Methods for general and molecular bacteriology. Amer Soc Microbiol 12:607–654. https://doi.org/10.3390/su11123393

29. Brenner DJ, Krieg NR, Staley JT, Garrity GM (2005) Bergey's manual of systematic bacteriology, 2nd Ed, Vol 2 (The Proteobacteria), part C (The Alpha-, Beta-, Delta-, and Epsilonproteobacteria) Springer New York
30. Vincent JM, Humphrey B (1970) Taxonomically significant group anti gens in Rhizobium. Microbiology 63:379–382. https:// doi. org/ 10. 1099/00221287-63-3-379
31. Kjeldahl, J. (1883). "Neue Methods zurBestimmung des Stickstoffs in organischenkorpern" (New method for the determination of nitrogen in organic substances), ZeitschriftfüranalytischeChemie, 22, 366-383.
32. Soltanpour PN (1985) Use of ammonium bicarbonate DTPA soil test to evaluate elemental availability and toxicity. Commun Soil Sci Plant Anal 16:323–338. https:// doi. org/ 10. 1080/ 00103 62850 9367607
33. Havlin JL, Soltanpour PN (1981) Evaluation of the NH4HCO3-DTPA soil test for iron and zinc. Soil Sci Soc Am J 45:70–75. https:// doi.org/10.2136/sssaj1981.03615995004500010016x
34. Walkley, A. 1947. A critical examination of a rapid method for determining organic carbon in soils:
35. Jackson, M. L. 1973. Soil Chemical Analysis. Prentic Hall (India) Pvt. Ltd. New Delhi.
36. Van-Loon, L. C. (2007). Plant responses to plant growth-promoting rhizobacteria. New perspectives and approaches in plant growth-promoting Rhizobacteria research, 243-254.
37. Baghaee R. S., & Heidarzadeh, N. (2014). Isolation and characterization of rhizosphere auxin producing Bacilli and evaluation of their potency on wheat growth improvement. Archives of Agronomy and Soil Science, 60(7), 895-905.
38. García-Fraile, P., Menéndez, E., & Rivas, R. (2015). Role of bacterial biofertilizers in agriculture and forestry. Aims Bioengineering, 2(3), 183-205.
39. Kumar, U., Panneerselvam, P., Gupta, V. V., Manjunath, M., Priyadarshinee, P., Sahoo, A., ... & Annapurna, K. (2018). Diversity of sulfur-oxidizing and sulfur-reducing microbes in diverse ecosystems. Advances in Soil Microbiology: Recent Trends and Future Prospects: Volume 1: Soil-Microbe Interaction, 65-89.
40. Armada, E., Roldán, A., & Azcon, R. (2014). Differential activity of autochthonous bacteria in controlling drought stress in native Lavandula and Salvia plants species under drought conditions in natural arid soil. Microbial ecology, 67, 410-420.

41. Vessey, J. K. (2003). Plant growth promoting rhizobacteria as biofertilizers. Plant and soil, 255, 571-586.
42. Nadeem, S. M., Ahmad, M., Zahir, Z. A., Javaid, A., & Ashraf, M. (2014). The role of mycorrhizae and plant growth promoting rhizobacteria (PGPR) in improving crop productivity under stressful environments. Biotechnology advances, 32(2), 429-448.
43. Marschner, P., Crowley, D., & Rengel, Z. (2011). Rhizosphere interactions between microorganisms and plants govern iron and phosphorus acquisition along the root axis–model and research methods. Soil Biology and Biochemistry, 43(5), 883-894.
44. Fitter, A. H., Helgason, T., & Hodge, A. (2011). Nutritional exchanges in the arbuscular mycorrhizal symbiosis: implications for sustainable agriculture. Fungal Biology Reviews, 25(1), 68-72.
45. Wu, S., Jia, S., Sun, D., Chen, M., Chen, X., Zhong, J., & Huan, L. (2005). Purification and characterization of two novel antimicrobial peptides subpeptin JM4-A and subpeptin JM4-B produced by Bacillus subtilis JM4. Current microbiology, 51, 292-296.
46. Adeleke, R., Nwangburuka, C., & Oboirien, B. (2017). Origins, roles and fate of organic acids in soils: A review. South African Journal of Botany, 108, 393-406.
47. Husson, O. (2023). How pH and Eh influence soil nutrient dynamics with microbial mediation. In Biological Approaches to Regenerative Soil Systems (pp. 221-238). CRC Press.
48. Snakin, V. V., Prisyazhnaya, A. A., & Kovács-Láng, E. (2001). Soil liquid phase composition. Elsevier.
49. Zhang, X., Zhang, S., Liu, S., Ren, D., & Zhang, X. (2023). Study on the migration behavior of heavy metals at the improved mine soil-plant rhizosphere interface. Environmental Technology, 1-26.
50. Boer, W. D., Folman, L. B., Summerbell, R. C., & Boddy, L. (2005). Living in a fungal world: impact of fungi on soil bacterial niche development. FEMS microbiology reviews, 29(4), 795-811.
51. Barrow, N. J., & Hartemink, A. E. (2023). The effects of pH on nutrient availability depend on both soils and plants. Plant and Soil, 1-17.

52. Boschetti, N. G., Quintero, C. E., & Giuffre, L. (2009). Phosphorus fractions of soils under Lotus corniculatus as affected by different phosphorus fertilizers. Biology and fertility of soils, 45, 379-384.

53. Khan, K.S., & Joergensen, R.G. (2009). Changes in microbial biomass and P fractions in biogenic household waste compost amended with inorganic P fertilizers. Bioresource Technology, 100(1), 303-309.

54. Behera, B. C., Mishra, R. R., Dutta, S. K., & Thatoi, H. N. (2014). Sulphur oxidising bacteria in mangrove ecosystem: A.

55. Rengel, Z. (2015). Availability of Mn, Zn and Fe in the rhizosphere. Journal of soil science and plant nutrition, 15(2), 397-409.

470

 Click here to access/download;Figure;Rabia Tables for PLOS ONE.docx

Table 1. Effect of microbial inoculants and fertilizer application on crop growth parameters

Treatment	Shoot length (cm)	Root length (cm)	Fresh shoot Weight (g)	Dry shoot weight (g)	Fresh root weight (g)	Root dry weight (g)
Control	75.9±2.96	17.3±1.86	11.4±1.32	8.00±0.10	1.31±0.03	0.90±0.10
1/2 NPK	84.1±2.33	19.1±1.18	19.9±1.08	10.2±0.94	1.93±0.07	1.39±0.16
full NPK	90.5±0.80	20.6±1.64	23.8±3.43	13.0±0.94	2.26±0.05	1.61±0.17
1/2 NPK+SOB	94.9±1.82	23.6±1.52	25.6±1.96	14.4±0.93	2.41±0.29	1.83±0.16
1/2 NPK+SRB	90.9±1.96	22.7±1.01	21.8±1.39	12.2±1.43	2.32±0.05	1.68±0.04
1/2 NPK+SOB+SRB	100±0.88	26.8±1.31	28.6±1.49	15.9±0.42	2.58±0.53	2.03±0.25

Each value is a mean of three replicates ± SD (n=3). Different letters indicate significant differences between treatments within columns by LSD test ($P \leq 0.05$). 1/2NPK, synthetic fertilizer @ 50–30-25 kg ha^{-1}; NPK, synthetic fertilizer @ 110–60-50 kg ha^{-1}; SOB, S-oxidizing bacteria; SRB, S-reducing bacteria

Table 2. Effect of microbial inoculants and fertilizer application on plant macronutrients

Treatments	Nitrogen (%)	Potassium (%)	Phosphorous (%)	Sulfur (%)
Control	0.67±0.055	0.95±0.024	1.79±0.065	0.043±0.015
1/2 NPK	1.08±0.088	1.06±0.027	2.02±0.090	0.056±0.038
Full NPK	1.42±0.140	1.49±0.024	2.31±0.026	0.062±0.110
1/2 NPK+SOB	1.27±0.196	1.41±0.022	2.54±0.044	0.180±0.106
1/2 NPK+SRB	1.18±0.056	1.32±0.023	1.85±0.052	0.110±0.086
1/2 NPK+SOB+SRB	1.53±0.065	1.44±0.038	2.80±0.070	0.210±0.026

Each value is a mean of three replications ± SD. Different alphabets show significant differences between treatments within columns by LSD test (P≤0.05). 1/2NPK, synthetic fertilizer @ 50–30-25 kg ha^{-1}; NPK, synthetic fertilizer @ 110–60-50 kg ha^{-1}; SOB, S-oxidizing bacteria; SRB, S-reducing bacteria

Table 3. Effect of microbial inoculants and fertilizer application on plant micronutrients

Treatment	Cu in Plant (mg kg^{-1})	Mn in plant (mg kg^{-1})	Fe in plant (mg kg^{-1})	Zn in plant (mg kg^{-1})
Control	4.66±0.05	29.7±4.3	146±7.22	28.8±0.53
1/2 NPK	5.36±0.15	34.3±3.5	171±14.7	32.6±2.55
full NPK	6.40±0.40	39.0±3.3	190±5.57	36.1±1.13
1/2 NPK+SOB	8.86±0.96	54.2±1.3	261±1.94	43.3±3.05
1/2 NPK+SRB	8.23±0.20	55.1±3.8	243±20.2	40.8±1.09
1/2 NPK+SOB+SRB	9.20±0.26	60.0±3.3	283±0.61	45.1±2.51

Each value is a mean of three replications ± SD. Different alphabets show significant differences between treatments within columns by LSD test (P≤0.05). 1/2NPK, synthetic fertilizer @ 50–30-25 kg ha^{-1}; NPK, synthetic fertilizer @ 110–60-50 kg ha^{-1}; SOB, S-oxidizing bacteria; SRB, S-reducing bacteria

Table 4. Effect of microbial inoculants and fertilizer application on soil chemical parameters

Treatment	Soil pH	EC (dSm^{-1})	Organic matter (%)
Control	7.5±0.09	0.251±0.02	0.50±0.16
1/2 NPK	7.5±0.11	0.283±0.03	0.55±0.19
full NPK	7.5±0.16	0.371±0.02	0.61±0.09
1/2 NPK+SOB	7.1±0.12	0.419±0.02	0.50±0.16
1/2 NPK+SRB	7.5±0.04	0.401±0.04	0.55±0.19
1/2 NPK+SOB+SRB	7.2±0.04	0.492±0.08	0.61±0.19

Each value is a mean of three replications ± SD. Different alphabets show significant differences between treatments within columns by LSD test ($P \leq 0.05$). 1/2NPK, synthetic fertilizer @ 50–30-25 kg ha^{-1}; NPK, synthetic fertilizer @ 110–60-50 kg ha^{-1}; SOB, S-oxidizing bacteria; SRB, S-reducing bacteria

Table 5. Effect of microbial inoculates and fertilizer application on soil macronutrients

Treatments	N (%)	P (mg kg^{-1})	K (mg kg^{-1})	SO_4 (mg kg^{-1})
Control	0.56±0.06	5.73±2.00	131±1.84	6.73±0.18
1/2 NPK	1.13±0.12	9.87±0.31	142±1.12	7.01±0.25
full NPK	1.32±0.21	17.7±1.90	151±3.15	7.72±0.41
1/2 NPK+SOB	1.49±0.09	21.0±1.06	156±1.55	12.7±0.85
1/2 NPK+SRB	1.12±0.06	14.7±4.46	149±5.97	10.8±1.01
1/2 NPK+SOB+SRB	1.53±0.07	24.5±3.00	154±3.21	13.3±0.76

Each value is a mean of three replications ± SD. Different alphabets show significant differences between treatments within columns by LSD test ($P \leq 0.05$). 1/2NPK, synthetic fertilizer @ 50–30-25 kg ha^{-1}; NPK, synthetic fertilizer @ 110–60-50 kg ha^{-1}; SOB, S-oxidizing bacteria; SRB, S-reducing bacteria

Table 6. Effect of microbial inoculates and fertilizer application on soil micronutrients

Treatments	Cu (mg kg^{-1})	Mn (mg kg^{-1})	Fe (mg kg^{-1})	Zn (mg kg^{-1})
Control	0.21±0.01	0.79±0.12	1.22±0.68	0.65±0.04
1/2 NPK	0.22±0.06	1.01±0.09	1.37±0.12	0.73±0.04
Full NPK	0.27±0.04	1.20±0.22	1.78±0.20	0.94±0.01
1/2 NPK+SOB	0.33±0.04	1.37±0.28	2.41±0.77	1.25±0.05
1/2 NPK+SRB	0.30±0.01	1.36±0.42	1.99±1.05	1.17±0.13
1/2 NPK+SOB+SRB	0.32±0.02	1.49±0.09	2.54±0.44	1.33±0.09

Each value is a mean of three replications ± SD. Different alphabets show significant differences between treatments within columns by LSD test ($P \leq 0.05$). 1/2NPK, synthetic fertilizer @ 50–30-25 kg ha^{-1}; NPK, synthetic fertilizer @ 110–60-50 kg ha^{-1}; SOB, S-oxidizing bacteria; SRB, S-reducing bacteria

Click here to access/download
Supporting Information
Declaration of Interest Statement.docx
