# Supplementary material for: Redox cycling of sulfur via microbes in soil boosts the bioavailability of nutrients to Brassica napus
Source: PLoS One. 2025 Feb 24;20(2):e0318936. doi: 10.1371/journal.pone.0318936 (PMC11849842; doi:10.1371/journal.pone.0318936)
Supplement: Attachment — Submitted filename: Response to Reviewers.docx [file pone.0318936.s003.docx]

**Response to Reviewers’ Comments**

**Reference:** PONE-D-24-23262

**Title:** Redox cycling of sulfur via microbes in soil boosts the bioavailability of nutrients to *Brassica napus*

**NOTE**: Revised/added text as per review comments has been highlighted in the revised manuscript through track changes.

Authors acknowledge the comments by respectable editor and reviewers to improve the quality of the manuscript. Thank you very much for these efforts and for sparing valuable time.

**Reviewer #1:**

**Query 1.** In the abstract numerical details are required.

**Response:** Numerical data has been added in the abstract as per suggestion.

**Query 2.** Keywords: Alphabetical rearrangement is suggested.

**Response:** Keywords have been arranged in alphabetical order as suggested.

**Query 3.** Line 58-62: simple, lucid, and complete sentences will be appreciated. Statement of novelty needed.

**Response:** Needful has been done and a statement of novelty is added in lines.

**Query 4.** Provide analysis of soil/characterization and plant analysis. The number of treatment combinations, design, and replications need to be disclosed in statistics.

**Response:** The initial data related to soil have been provided under subheading 2.1 Soil characterization. Plant analysis has been described under subheading 2.9 Plant analysis. The number of treatment combinations, design, and number of replications has been added as suggested under subheading 2.10 Statistical analysis however, the same information is available in subheading, 2.6 Treatment plan, and experimental design

**Query 5.** Authors are required to include (i) how the farmers will benefit from the study and (ii) the consequences of excess S application on the PK cycle.

**Response:** Suggestions have been incorporated in the last paragraph of the discussion.

**Query 6.** Table 1. Effect of microbial inoculants and fertilizer application on crop growth parameters. Table 2-6. Effect of microbial inoculants and fertilizer application on plant macronutrients. Data should be accompanied by letter cases. Some data should be in figure format.

**Response:** All tables include alphabets showing significant differences. Additionally, the plant macronutrient data (Table 2) has been converted into a graph (Fig 1), as suggested.

**Query 7.** English check suggested.

**Response:** Needful has been done.

**Reviewer #2:**

**Query 1.** Line 23-23: In the first phase isolation of S bacteria was carried out while in the second phase, results are missing.

**Response:** Data related S bacteria has been provided under subheading 3.1 SOB and SRB isolate attributes. In this step, only characterization of isolates was carried out.

**Query 2.** KSO4^-2^

**Response:** Line 173 corrected the typing error K_2_SO_4_.

**Query 3.** The conclusion should be concrete. (% increase in yield and nutrient availability with best treatment).

**Response:** Suggestion incorporated.

**Query 4.** Isolation and Characterization of SOB and SRB, results not given.

**Response:** Data has been provided under subheading 3.1 SOB and SRB isolate attributes.

**Query 5.** All the references have been updated and formatted as the journal format.

**Response:** Needful has been done.

**Query 6.** Alphabets showing significant differences are missing in the table

**Response:** Alphabets showing significant differences are included in all tables and Figure as per suggestion.

**Reviewer #3:**

**Query 1.** Example of repetition: In the paragraph discussing the importance of sulfur for plant nutrition, there is a repetition of ideas about sulfur’s role in proteins and photosynthesis. The text could be more concise, addressing these points more directly.

**Response:** Suggestion incorporated.

**Query 2.** Example of critical analysis: The discussion mentions that the inoculation of SOB and SRB increased nutrient bioavailability, but it does not address comparisons with other studies that may show different results.

**Response:** Although the related data is very limited, however, a few relevant data are included in the discussion section.

**Query 3.** Example of grammatical error: In some parts, some phrases could be clearer. For example, in a section:

"The applicability of the results obtained are evident to increase agricultural productivity." The correct form would be: "The applicability of the results obtained is evident in increasing agricultural productivity." Additionally, phrases like "The effect of SOB and SRB on plants were significant" should be revised to "The effect of SOB and SRB on plants was significant," correcting the subject-verb agreement error.

**Response:** Suggestion incorporated.

**Query 4.** Supporting Data:

Example of raw data: If the authors mention that SOB and SRB inoculation increased nutrient concentrations like nitrogen (N) and phosphorus (P), it would be helpful to include the raw numeric values, such as: "The nitrogen concentration in the plant tissue was 1.53% in the ½NPK+SOB+SRB combination." These data should be presented in more detail, perhaps in a supplemental table or a publicly accessible data repository.

**Response:** Needful has been done.

**Query 5.** The conclusion could be more detailed.

**Response:** The conclusion has been updated as per suggestion.
